# Long lasting effect of acute restraint stress on behavior and brain anti-oxidative status

**DOI:** 10.3934/Neuroscience.2022005

**Published:** 2022-01-27

**Authors:** Nouhaila Chaoui, Hammou Anarghou, Meriem Laaroussi, Oumaima Essaidi, Mohamed Najimi, Fatiha Chigr

**Affiliations:** Biological Engineering Laboratory, Faculty of Sciences and Techniques, Sultan Moulay Slimane University, Beni Mellal, Morocco

**Keywords:** restraint stress, acute stress, anxiety, behavior, oxidative stress

## Abstract

Exposure to certain acute stressors results in an immediate behavioral and physiological response to these situations during a significant period of days. The goal of the current study is to evaluate the long-lasting effect of single exposure of restraint stress among mice after 0 h, 24 h, 48 h and 72 h. Five groups of mice are under experiment: a control group and four groups exposed to one session of restraint stress. All these groups have been studied for behavioral tests in order to evaluate their memories. This is done through a Y-labyrinth and an object recognition test, and anxiety by using open field device. In the second part of the study, enzymatic assays (concerning catalase, glutathione s transferase, glutathione peroxidase and superoxide dismutase) are used to evaluate oxidative stress. The enzymatic activity of the antioxidant system is assessed in five brain structures, including the cerebellum, olfactory bulb, spinal bulb, hypothalamus, and hippocampus.

The obtained results show that acute restraint stress leads to a decrease in memory function and to the development of an anxious state; concomitant to an increase of locomotor activity afterword. It causes disturbance of antioxidant balance in the brain by developing a state of oxidative stress. Indeed, restraint stress causes a change in anti-oxidant stress enzymatic activity in the brain, notably in post-stress period. In conclusion, acute restraint stress is responsible for altering cognitive functions, especially memory, and the development of anxious behavior, which could be a result of the generation of oxidative stress; effects that are persistent over an important period after the cessation of stress.

## 1. Introduction

Stress is well established as a serious health problem as it causes many concerns leading in some cases to major pathologies such as diabetes [1], cardiovascular failure [2],[3], cancer [4] and depression [5]. Other emerging deleterious effects of stress include feeding disorders and body weight (BW) dysregulations [6],[7]. Concerning animals, many studies reported that different paradigms of stress produce a significant reduction in food intake (FI) unless access to food is given after the stress period. On the other hand, some studies report that among humans, psychological stress paradigms are more effective in producing such manifestations [8]–[11]. It is well admitted now that psychological stress could lead to the increase of stress hormones (e.g., cortisol, epinephrine) but also cytokines (e.g., interleukin [IL]-1, IL-6, tumor necrosis factor [TNF]-α) in its immune component [12]. Depending on stress intensity and duration, this rise in stress hormones and cytokines could persist after the cessation of the stress session [13],[14] as for other regulatory factors [9],[15]. Interestingly, psychological stress elicits also a multitude of cellular events that could be harmful to the integrity of the cell,notably in the case of neurons. Indeed, psychological stress activates reactive oxygen species: ROS gets generated in the brain [16]. From these ROS, we can cite superoxide ($O_{2}^{-}$), hydrogen peroxide (H_2_O_2_), and hydroxyl radical (•OH). ROS accumulation occurs due to an imbalance between ROS generative and scavenging activities in the cell [17],[18]. To counteract these negative effects, the cell machinery possesses highly efficient ROS scavenging strategies in organelles. This is the case of mitochondria containing high levels of superoxide dismutase involved in the conversion of the toxic $O_{2}^{-}$ into either O_2_ or H_2_O_2_ [18].

At this point, another enzyme takes over by dissociating hydrogen peroxide H_2_O_2_ into dioxygen O_2_ and water H_2_O in order to annihilate its toxic effect. Other cellular organelles like ER engages antioxidation pathways involving glutathione reductase. It is now admitted that oxidative stress toxicity occurs when the capacity of scavenging is not enough to alleviate the harmful effects of ROS leading to a cellular dysfunction [18]–[20]. As it has been previously suggested, there is a close relation between psychological stress and generation of oxidative stress. Indeed, the activation of the hypothalamic–pituitary–adrenal axis under psychological stress causes inflammatory oxidative stress in the brain [16]. If this has been well documented in chronic stress that results long-lasting impairments of brain functions, less is known about the long-lasting effects of acute psychological stress.

The goal of this study is to investigate this aspect based on previous data reporting that one stress session could elicit harmful effects (see above). For this, we used a psychological stress paradigm (restraint stress) and analyzed the temporal evolution of scavenging enzymes constituting the first frontline and those belonging to glutathione pathways forming the second line, up to 72 hours post stress. Simultaneously, we are also investigating behavioral aspects of stressed animals at the same experimental points to approach the eventual effects of generated oxidative stress on behavioral change. This will give another brick concerning the interplay among psychological stress, oxidative stress, and the generation of mental/neurological disorders.

## 2. Materials and methods

### 2.1. Animals and experimental design

Thirty Swiss albino male mice weighing approximately 30 g were used in this study. They were obtained from Animal husbandry of the Faculty of Sciences and Techniques, Beni Mellal, Morocco. The mice were divided into five groups, four stressed groups (n = 6 for each group) and a control group (n = 6). The four stressed groups consisted of mice sacrificed after 0 h, 24 h, 48 h and 72 h after the termination of a session of one hour (1 h) restraint stress. All the mice were housed on an ad libitum diet under a standard condition of temperature (22 °C) and a 12:12 h dark: light cycle. Experimental protocols were carried out in compliance with institutional Ethical Committee guidelines for animal research. The acute restraint stress consisted of placing the mice in flexible, transparent plastic cones with an opening at the bottom. These cones are 11.50 cm long and 3 cm in diameter. Mice were immobilized for one hour on a single exposure.

Behavioral tests were then conducted to assess the effect of stress on memory and anxiety. Mice were sacrificed by decapitation at 0 h, 24 h, 48 h or 72 h after stress; control mice were sacrificed at identical times of day for biochemical and enzymatic analyzes.

### 2.2. Behavioral tests

#### 2.2.1. Y maze

The Y labyrinth is used to assess short-term memory, general locomotor activity, and behavior. Consequently, the spontaneous alternation was evaluated using a Y labyrinth composed of three equally spaced arms (120 °C, 41 cm long and 15 cm high). The floor of each arm is made of Plexiglas and it is 5 cm wide. Each mouse was placed in one of the three arms of the device to record their behaviors.

For this test of spontaneous alternation, the animal is placed in one of the arms and then left for 5 minutes. Its activity is recorded using a camera. The arms are labeled A, B and C to evaluate the sequence of entries in the different arms. At the same time, we calculate the percentage of alternation. The change can be perceived as the consecutive entry into the three arms of the device.

#### 2.2.2. Novel object recognition test (NORT)

The object recognition test is particularly useful for studying declarative memory among rodents because it appeals to their natural preference for a new object compared to a familiar one. This task evaluates the ability of a mouse to recognize a new object compared to a familiar one in a known environment. Control animals typically spend more time exploring the new object, which reflects the use of memory and learning processes. The absence of difference in the exploration of objects can be interpreted as a memory deficit. In most of the protocols, the test is divided into 3 phases: a familiarization phase (the animal is free to explore the space without the presence of objects), one or several phases of training (the animal is in the presence of two similar objects) and a test phase (the experimenter introduces a new object instead of a familiar one).

At the very beginning, the animal is placed in the device for familiarization. Then it is given two objects A and B which are similar (A = B), to explore them for 5 mins. After that, we put it back in its original cage for 2 hours. We replace the object B with a different object C in the form and the color. Then, we put the animal in the device to see how it will explore the new object for a period of 5 mins.

#### 2.2.3. Open field

This test analyzes the rodent's exploratory behavior in a confined space. It is used primarily to measure motor functions, as well as to evaluate the anxiety level. An anxious animal avoids the open ground center and remains near the walls.

Recording its behavior by using a camera, the animal is placed to the center of the device, and left for 5 mins. The calculated parameters are the time spent at the center, in the periphery, and the squares crossed at the center, the periphery, and the locomotor activity.

### 2.3. Biochemical assays

#### 2.3.1. Procedures for brain and tissue dissection

Mice were anesthetized with pentobarbital and then decapitated. After that, the brains of all the groups were removed from the skull and recovered. Each brain, including the cerebellum, olfactory bulb, spinal bulb, hypothalamus, and hippocampus, were rapidly dissected on a plate at 4 °C, weighed, and frozen at −20 °C until use for the biochemical assays.

#### 2.3.2. Tissue preparation

Brain structures were removed immediately and homogenized in buffer solution TBS (50 mM Tris, 150 mM NaCl, pH 7.4); homogenates were centrifuged at 10,000×g for 15 min at 4 °C. The supernatant was used for Acetylcholinesterase, Malondialdehyde, Catalase, Superoxide Dismutase, Glutathione S Transferase, and Glutathione Peroxidase.

#### 2.3.3. Determination of acetylcholinesterase activity

Acetylcholinesterase (AChE)-specific activity was measured according to the method of Ellman et al. [21] using acetylthiocholine iodide as a substrate. The reaction mixture contained phosphate buffer (0.1 M, pH 8.0), acetylthiocholine iodide (0.075 M), and 5, 5dithiobis-2-nitrobenzoic acid (DTNB; 0.01 M). After adding the brain structure tissue homogenate, the hydrolysis rate of acetylcholine iodide was measured by a spectrophotometer at 412 nm. The enzyme activity was expressed as µmol Ach hydrolyzed/min/mg of protein.

#### 2.3.4. Determination of lipid peroxidation

The lipid peroxidation (LPO) level in the brain was measured by the method of Buege and Aust [22]. In this experiment, a total of 125 µl of supernatant was homogenized with 50 µl of PBS and 125 µl of 20% TCA + 1% BHT (TCA-BHT), to precipitate proteins, and centrifuged (1000×g, 10 min, 4 °C). Afterwards, 200 µl of supernatant was mixed with 40 µl of HCl (0.6 M) and 160 µl of TBA dissolved in Tris (120 mM). The mixture was heated at 80 °C for 10 min, and the absorbance was measured at 530 nm. The amount of thiobarbituric acid reactive substances (TBARS) was calculated using a molar extinction coefficient of 1.56 × 105 M/Cm.

#### 2.3.5. Catalase

The activity of catalase (CAT) was measured at 240 nm using a UV/visible spectrophotometer by the change in optical density due to the disproportionation of hydrogen peroxide (H) [23]. For the reaction, we added 20 µl of supernatant to 780 µl of phosphate buffer saline (PBS) (0.1 M; pH 7.4) and 200 µl of H_2_O_2_.

#### 2.3.6. Glutathione peroxidase

For each assay, a mixture containing 200 µl of supernatant, 200 µl of phosphate buffer (100 mM), 200 µl of 4 mM GSH, and 400 µl of H_2_O_2_ (5 mM) was incubated for 1 min at 37 °C. After adding 500 µl of 5% TCA, the mix was then centrifuged at 1500×g for 5 min. 200 µl of the supernatant was recovered, we added 500 µl of phosphate buffer and 500 µl of DTNB, and the absorbance was measured at 412 nm each min for 5 min according to a modified method of Flohe and Gunzler [24].

#### 2.3.7. Glutathione s-transferase activity

The Glutathione S-transferase activity (GST) activity was determined by the method of Habig and Pabst [25]; The assay of GST activity is based on a substrate, usually 1-chloro-2,4-dinitrobenzene (CDNB), which reacts readily with many forms of GST and glutathione. The conjugation reaction of these two compounds results in the formation of a new molecule that absorbs light at 340 nm wavelength. The mixture contained 830 ml of phosphate buffer (0.1 M; pH 6.5), 50 ml of CDNB, 100 ml of GSH 20 ml of homogenate.

#### 2.3.8. Superoxide dismutase

Superoxide dismutase (SOD) activity was determined by the method of Asada et al. [26]; 0.05 ml of the supernatant was added to 0.1 ml of a mixture containing methionine (13 mM) and Na EDTA (0.1 mM), 0.8922 ml of phosphate buffer (50 mM, pH = 7.8), 0.95 ml of phosphate buffer, 0.088 ml of NBT (2.64 mM), and 0.0226 ml of riboflavin (0.26 mM). The reduction of NBT was estimated after 20 min at a wavelength of 580 nm.

#### 2.3.9. Protein measurement

Protein concentration was assayed by the method of Lowry et al. [27] with bovine serum albumin as the standard.

### 2.4. Statistical analysis

All data were expressed as mean ± *SEM*. All data were analyzed by ordinary one-way ANOVA followed by Tukey's post hoc test for multiple comparisons. All analyses were performed with Graph pad prism 9. Differences were considered significant at a p-value *p < 0.05 compared with the control group.

## 3. Results

### 3.1. Y maze test

**Figure 1.**
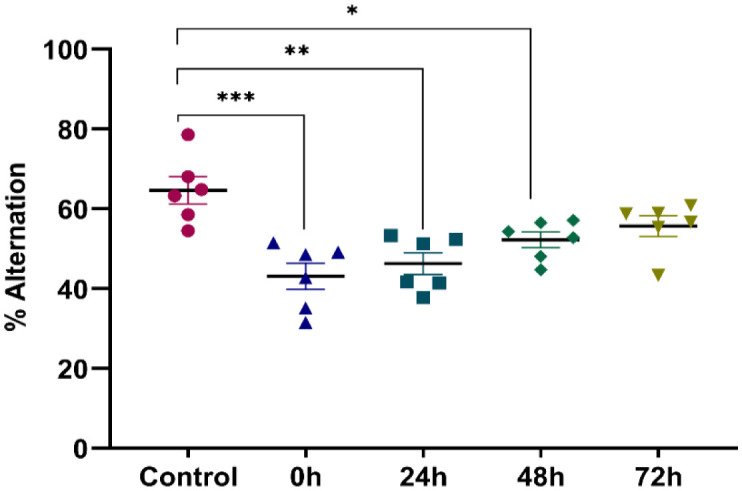
Effect of stress on alternation performance of mice obtained by the Y maze test in the different groups of the experiment. Values represent the mean ± *SEM*. (One Way ANOVA test followed by Tukey test : *p < 0.05 compared to the control *F* (4, 25) = 8.664.

In this test, we evaluated the effect of restraint stress on the spontaneous alternation of mice. A significant decrease in the alternation percentage was observed among the four stressed groups compared to control (Control: 64.655 ± 3.401, 0 h: 43.105 ± 3.336, 24 h: 46.298 ± 2.761, 48 h: 52.262 ± 2.006, 72 h: 55.662 ± 2.588, *F* (4, 25) = 8.664; *P* = 0.0002) (Figure 1). This decrease was more accentuated in both the 0 h and 24 h groups. Stressed mice have a lower percentage of alternation than the control ones.

### 3.2. Novel object recognition test

**Figure 2.**
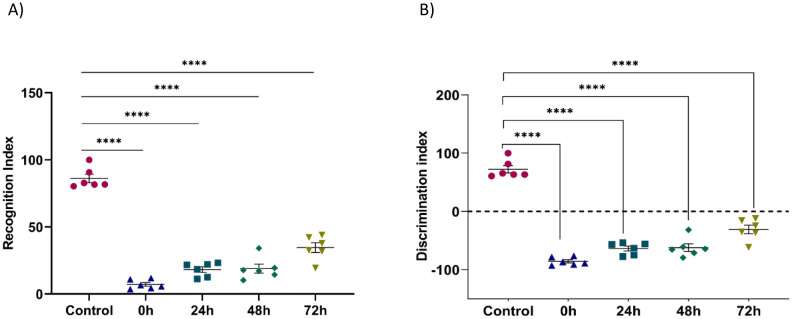
Effect of restraint stress on (A) Recognition index and B) Discrimination index obtained by the Object Recognition Test in the different groups of the experiment. Each value represents the mean ± *SEM*. One Way ANOVA test followed by Tukey test: *p < 0.05 compared to the control group *F* (4, 25) = 120.3.

In this test, we assessed the effects of stress on the exploration time of two objects, A and B, and on the recognition index in stressed mice and their controls. The results obtained in this test showed that the time spent exploring the objects (familiar and novel) differed significantly between the five groups. Furthermore, the stressed groups demonstrated a significantly lower object recognition index compared to the control group (Control: 86.19 ± 3.155;0 h: 7.228 ± 1.401, 24 h: 18.21 ± 2.095, 48 h: 18.92 ± 3.319, 72 h: 34.6 ± 3.654, *F* (4, 25) = 120.3; *P* < 0.0001). (Figure 2-A). The percentage of discrimination index of the stressed was significantly lower compared to the control mice (Control: 72.38 ± 6. 309, 0 h: −85.54 ± 2.802, 24 h: −63.58 ± 4.190, 48 h: −62.15 ± 6.637, 72 h: −30.8 ± 7.307, *F* (4, 25) = 120.3; *P* < 0.0001) (Figure 2-B).

Controls spent more time exploring the novel object, while stressed mice spent more time exploring the familiar object. In fact, the stressed mice had a worse performance in the exploration of the familiar object compared to controls. We concluded that stress had an effect on recognition memory.

### 3.3. Open field test

**Figure 3.**
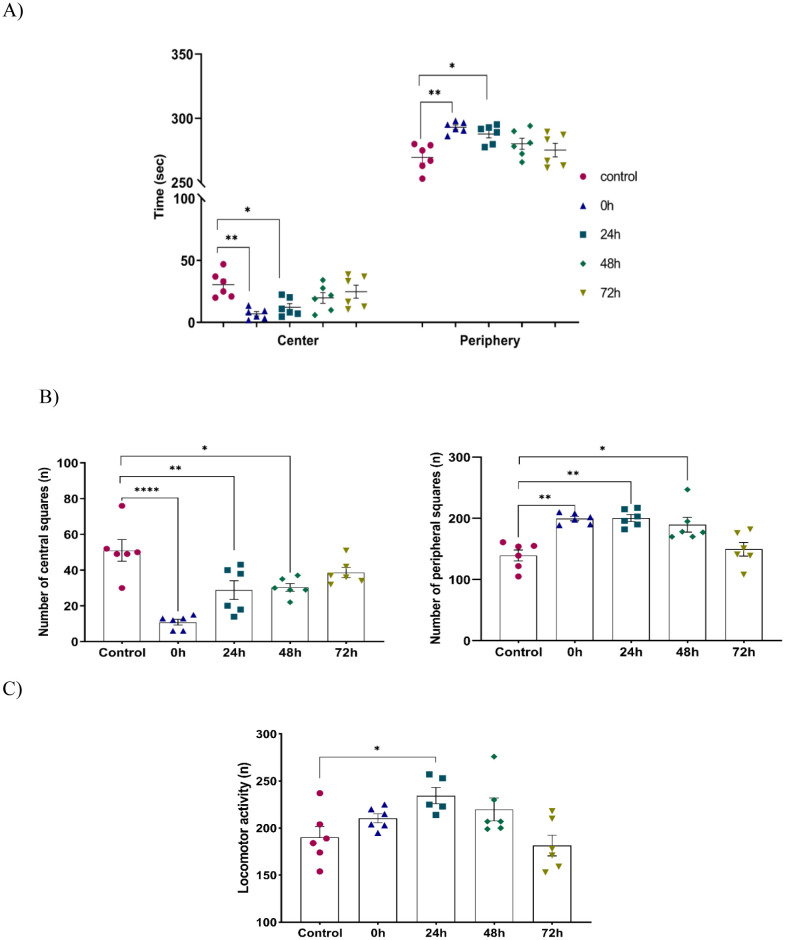
Behavior in the open field during the 5 min of recording. Each histogram represents the mean ± *S.E.M*. A) Time spent in center and periphery F (4, 25) = 5.827. B) Number of crossed squares; central squares: *F* (4, 25) = 13.80, peripheral squares: *F* (4, 25) = 10.73. C) locomotor activity *F* (4, 24) = 4.397. Each value represents the mean ± *SEM*. One Way ANOVA test followed by Tukey test: *p < 0.05, compared to the control group.

The analysis of the results of Open Field test showed an effect of stress on the time and the number of crossed squares. Thus, a significant increase in the spent time at the periphery by the stressed groups is equivalent to the decrease in the time spent in the center by the same groups compared to the control group (Center: control: 30.500 ± 4.288, 0 h: 6.938 ± 1.787, 24 h :12.275 ± 2.979, *F* (4, 25) = 5.827; *P* = 0.0019) (Periphery: control: 269.500 ± 4.288, 0 h: 293.062 ± 1.787, 24 h: 287.725 ± 2.979, *F* (4, 25) = 5.827; *P* = 0.0019) (Figure 3-A).

Furthermore, the number of peripheral squares crossed was higher among the stressed animals compared to the controls (control: 139.333 ± 8.962, 0 h: 199.500 ± 3. 612, 24 h: 200.500 ± 5.661, 48 h: 189.500 ± 12.091, *F* (4, 25) = 10.73; *P* < 0.0001). Whereas the number of central squares crossed by the controls was higher compared to stressed mice (control: 51.000 ± 5.989, 0 h: 10.833 ± 1.579, 24 h: 28.833 ± 5.244, 48 h: 30.333 ± 2.155, 72 h: 38.667 ± 2.824, *F* (4, 25) = 13.80; *P* < 0.0001) Figure 3–B). A comparison of mobility in the different groups shows that there is an increase among the stressed groups 24 h and 48 h (control: 190.333 ± 11.537, 24 h: 229.333 ± 8.682, 48 h: 219.833 ± 12.131, *F* (4, 24) = 4.397; *P* = 0.0083). (Figure 3-C).

### 3.4. Acetylcholinesterase activity «AChE»

**Figure 4.**
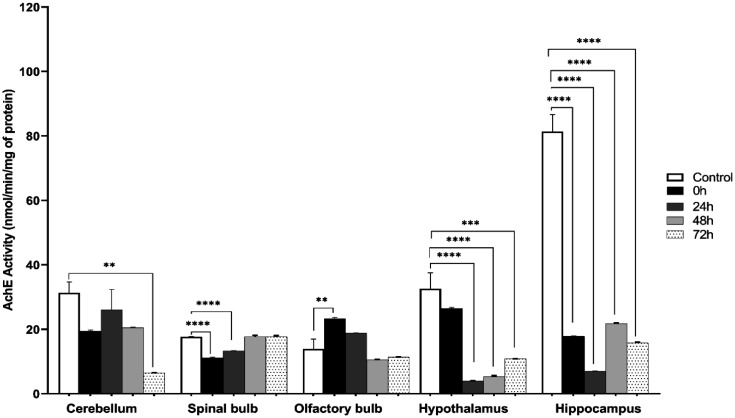
AChE activities in different brain structures cerebellum, The olfactory bulb, spinal bulb, hypothalamus, hippocampus of stressed mice and control. Specific activity was expressed as nmol ATC hydrolyzed/min/mg protein. (mean ± *SEM*, n = 6 per group. One Way ANOVA test followed by Tukey test: *p < 0.05 differs from control group). Cerebellum: *F* (4, 10) = 8.265, spinal bulb: *F* (4, 10) = 134, olfactory bulb: *F* (4, 10) = 15.29, hypothalamus: *F* (4, 10) = 33.37, hippocampus: *F* (4, 10) = 162.2.

Among the five brain structures analyzed of the stressed mice and their corresponding control, there was a significant decrease in AChE activity in the cerebellum of group 72 h (control: 31.326 ± 3.363, 72 h: 6.498 ± 0.0328; *F* (4, 10) = 8.265 ; *P* = 0.0033), spinal bulb (control:17.62 ± 3.053, 0 h: 11.164 ± 0.156, 24 h: 13.276 ± 0.0701; *F* (4, 10) = 134 ; *P* < 0.0001), hypothalamus (control: 32.577 ± 4.987, 24 h: 3.939 ± 0.127, 48 h: 5.366 ± 0.246, 72 h: 10.827 ± 0.00566; *F* (4, 10) = 33.37 ; *P* < 0.0001 ), hippocamus (control: 81.377 ± 5.242, 0 h: 17.867 ± 0.0623, 24 h: 7.035 ± 0.0501, 48 h: 21.803 ± 0.168, 72 h: 15.792 ± 0.209; *F* (4, 10) = 162.2; *P* < 0.0001). On the other hand, there was a significant increase in AChE activity in the olfactory bulb of 0 h group (control: 13.877 ± 3.053, 0 h: 23.337 ± 0.361; *F* (4, 10) = 15.29; *P* = 0.0003) (Figure 4).

### 3.5. Malondialdehyde activity MDA

To estimate brain lipid peroxidation, MDA activity was measured in five brain structures, namely: cerebellum, olfactory bulb, spinal bulb, hypothalamus, and hippocampus, of stressed and control mice.

**Figure 5.**
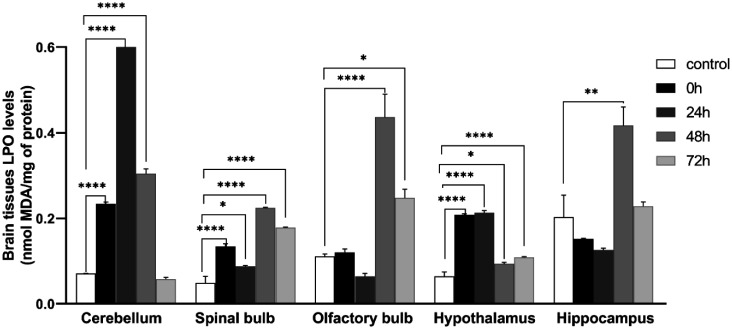
Lipid peroxidation (LPO) levels of brain tissue of mice stressed and there control. All data are presented as the mean ± *SEM*; *p < 0.05, **p < 0.01, ***p < 0.001, One Way ANOVA test followed by Tukey test: *p < 0.05 compared with the control group. Cerebellum: *F* (4, 10) = 1106, spinal bulb: *F* (4, 10) = 89.64, olfactory bulb: *F* (4, 10) = 33.08, hypothalamus: *F* (4, 10) = 166, hippocampus: *F* (4, 10) = 14.02.

The activity was significantly increased in all brain structures of stressed groups such as in the cerebellum (control: 0.0707 ± 0.000552, 0 h: 0.234 ± 0.00423, 24 h: 0.733 ± 0.0135, 48 h: 0.304 ± 0.0113; *F* (4, 10) = 1106 ; *P* < 0.0001), spinal bulb (control: 0.0494 ± 0.0148, 0 h: 0.134 ± 0.00635, 24 h: 0.088 ± 0.00233, 48 h: 0.224 ± 0.00142, 72 h: 0.178 ± 0.00166 ; *F* (4, 10) = 89.64 ; *P* < 0.0001), olfactory bulb (control: 0.111 ± 0.00571, 48 h: 0.4364 ± 0.0537, 72 h: 0.2477 ± 0.0201; *F* (4, 10) = 33.08 ; *P* < 0.0001, hypothalamus (control: 0.065 ± 0.00995, 48 h: 0.094 ± 0.0034; *F* (4, 10) = 166 ; *P* < 0.0001), hippocampus (control: 0.203 ± 0.0516, 48 h: 0.417 ± 0.0435; *F* (4, 10) = 14.02 ; *P* = 0.0004 (Figure 5).

### 3.6. Catalase activity

**Figure 6.**
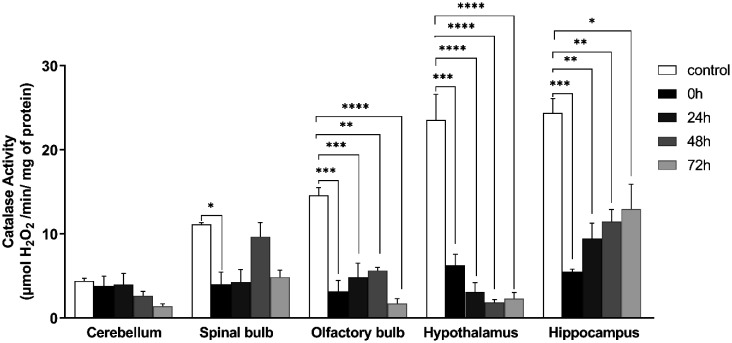
Variation of catalase activity in five brain structures: cerebellum, olfactory bulb, spinal bulb , hypothalamus, hippocampus in different groups of the experiment. Values are represented as mean ± *SEM*, (One Way ANOVA test followed by Tukey test : *p < 0.05). Cerebellum: *F*(4, 7) = 1.647, spinal bulb: *F*(4, 7) = 8.382, olfactory bulb: *F* (4, 10) = 21.90, hypothalamus: *F* (4, 10) = 33.27, hippocampus: *F* (4, 10) = 14.31.

The Catalase activity among the stressed groups showed a significant decrease in: spinal bulb (control: 4.401 ± 0.339, 0 h: 4.025 ± 1.440; *F*(4, 7) = 8.382 ; *P* = 0.027) , olfactory bulb (control: 11.140 ± 0.195, 0 h: 3.191 ±1.277, 24 h: 4.829 ± 1.681, 48 h: 5.654 ± 0.353, 72 h: 1.725 ± 0.575 ; *F* (4, 10) = 21.90; *P* < 0.0001), hypothalamus (control: 23.549 ± 3.034; 0 h: 6.299 ± 1.260, 24 h: 3.083 ± 1.111, 48 h: 1.843 ± 0.369, 72 h: 2.319 ± 0.714; *F* (4, 10) = 33.27; *P* < 0.0001), hippocampus (Control: 24.367 ± 1.742, 0 h: 5.521 ± 0.290, 24 h: 9.433 ± 1.858, 48 h: 11.480 ± 1.427, 72 h: 12.959 ± 2.974; *F* (4, 10) = 14.31 ; *P* = 0.0004.). A marked decrease was observed in hypothalamus and hippocampus (Figure 6).

### 3.7. Glutathione peroxidase activity

GPx activity showed contrasting effects in the different brain structures analyzed. We noticed a significant increase in the cerebellum of 24 h group (control: 0.00160 ± 0.0002, 24 h: 0.00427 ± 0.00019, *F* (4, 10) = 102; *P* < 0.0001), spinal bulb (control: 0.00036 ± 0.000277, 0 h: 0.00106 ± 0.0000217, 48 h: 0.00125 ± 0.00002; *F* (4, 10) = 7.112; *P* = 0.0056), olfactory bulb (control: 0.0014 ± 0.000383, 0 h: 0.00274 ± 0.0000853, *F* (4, 10) = 22.09; *P* < 0.0001), hypothalamus (control: 0.00056 ± 0.0000756, 0 h: 0.0064 ± 0.0000735, *F* (4, 10) = 895.2; *P* < 0.0001), hippocampus (0.00093 ± 0.0000161, 0 h:0.0015 ± 0.0000105, 24 h: 0.00156 ± 0.00000786; *F* (4, 10) = 9.374; *P* = 0.0020).

**Figure 7.**
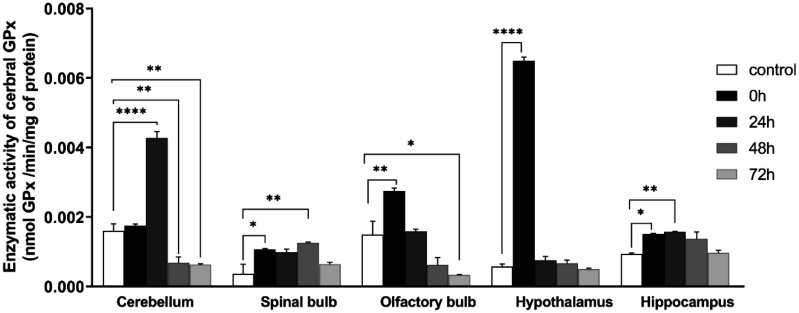
Variation of glutathione peroxidase activity in five brain structures: cerebellum, olfactory bulb, spinal bulb, hypothalamus, hippocampus in different groups of the experiment Values are represented as mean ± *SEM*, (One Way ANOVA test followed by Tukey test : *p < 0.05). Cerebellum, *F* (4, 10) = 102, spinal bulb: *F* (4, 10) = 7.112, olfactory bulb: *F* (4, 10) = 22.09, hypothalamus: *F* (4, 10) = 895.2, hippocampus: *F* (4, 10) = 9.374.

While there was a decrease in the cerebellum of the groups 48 h and 72 h (Control: 0.00160 ± 0.000200, 48 h: 0.00067 ± 0.000166, 72 h: 0.000626 ± 0.0000293, *F* (4, 10) = 102; *P* < 0.0001), olfactory bulb of the groups 48 h & 72 h (control: 0.0014 ± 0.000383, 48 h: 0.000621 ± 0.000212, 72 h: 0.000331 ± 0.0000117; *F* (4, 10) = 22,09; *P* < 0.0001) (Figure 7).

### 3.8. Glutathione S transferase activity

**Figure 8.**
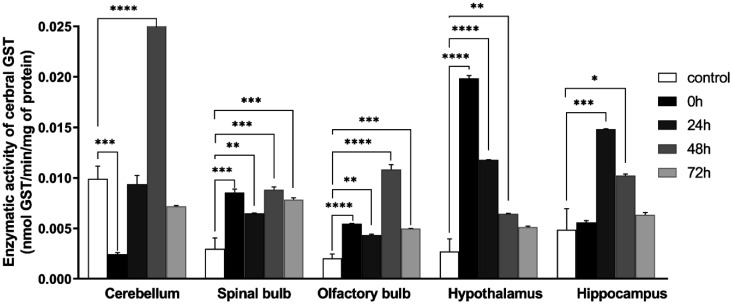
Variation of glutathione-S-transférase activity in: cerebellum, olfactory bulb, spinal bulb, hypothalamus, hippocampus in different groups of the experiment. Values are represented as mean ± *SEM*, (One Way ANOVA test followed by Tukey test: *p < 0.05). Cerebellum: *F* (4, 10) = 248.2, spinal bulb: *F* (4, 10) = 20.37, olfactory bulb: *F* (4, 10) = 122.1, hypothalamus: *F* (4, 10) = 144.7, hippocampus: *F* (4, 10) = 19.23.

The stress significantly increased glutathione S transferase activity among most of the brain structures: cerebellum of the group 48 h (control: 0.009 ± 0.00125, 48 h: 0.0309 ± 0.000368; *F* (4, 10) = 248.2; *P* < 0.0001), spinal bulb (control: 0.00298 ± 0.00108, 0 h: 0.00854 ± 0.000351, 24 h: 0.00650 ± 0.0000217, 48 h: 0.00884 ± 0.000277, 72 h: 0.00784 ± 0.000179, *F* (4, 10) = 20.37; *P* < 0.0001), olfactory bulb (control: 0.00203 ± 0.000434, 0 h: 0. 00546 ± 0. 0000472, 24 h: 0. 00433 ± 0.0000997, 48 h: 0.0108 ± 0.000481, 72 h: 0.00496 ± 0.0000149; *F* (4, 10) = 122.1; *P* < 0.0001), hypothalamus (control: 0.00271 ± 0.00124, 0 h: 0.0199 ± 0.000258, 24 h: 0.0118 ± 0.0000208, 48 h:0.00645 ± 0.0000260, 72 h: 0.00511 ± 0.000124, *F* (4, 10) = 144.7; *P* < 0.0001), hippocampus (control: 0.00487 ± 0.00209, 24 h: 0.0148 ± 0.0000416, 48 h:0.0102 ± 0.000178, *F* (4, 10) = 19.23; *P* = 0.0001). On the other hand, it is decreased in the cerebellum of the group 0 h (control: 0.009 ± 0.00125, 0 h: 0.00243604 ± 0.000167, *F* (4, 10) = 248.2; *P* < 0 .0001) (Figure 8).

### 3.9. Superoxide dismutase «SOD» activity

**Figure 9.**
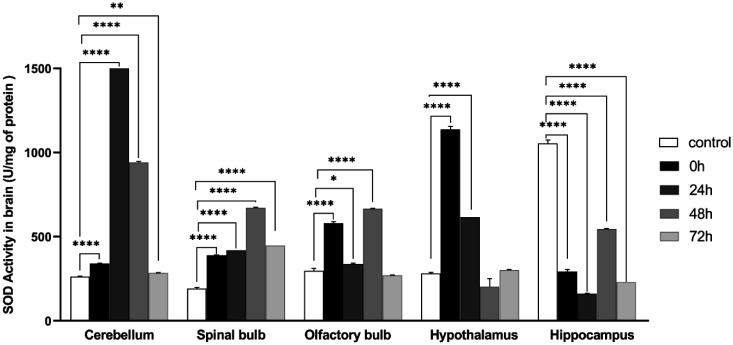
Variation of superoxide dismutase activity in cerebellum, olfactory bulb, spinal bulb, hypothalamus, hippocampus in different groups of the experiment Values are represented as mean ± *SEM*, (One Way ANOVA test followed by Tukey test: *p < 0.05). Cerebellum: *F* (4, 10) = 34388, spinal Bulb: *F* (4, 10) = 2726, olfactory Bulb: *F* (4, 10) = 448.1, hypothalamus: *F* (4, 10) = 264.3, hippocampus: *F* (4, 10) = 1177.

The activity of SOD is characterized by contrasting effects in the different brain structures analyzed. We noticed a significant increase in the cerebellum (control: 262.168 ± 2.545, 0 h: 340.779 ± 2.053, 24 h: 1614.170 ± 0.000, 48 h: 942.290 ± 5.964, 72 h: 284.376 ± 2.122, *F* (4, 10) = 34388; *P* < 0.0001), spinal bulb (control: 192.506 ± 5.298, 0 h: 389.834 ± 2.670, 24 h : 419.275 ± 0.000, 48 h: 671.606 ± 4.278, 72 h: 448.158 ± 0.000; *F* (4, 10) = 2726; *P* < 0.0001), olfactory bulb (control: 296.842 ± 14.695, 0 h: 579.493 ± 9.523, 24 h: 337.033 ± 5.897, 48 h: 665.596 ± 3.986, *F* (4, 10) = 448.1; *P* < 0.0001), hypothalamus (control: 280.78 ± 7.589, 0 h: 1136.76 ±18.797, 24 h: 615.39 ± 0; *F* (4, 10) = 264.3; *P* < 0,0001). Whereas there is a significant decrease in hippocampus (control: 1054.74 ± 19.652, 0 h: 292.48 ± 12.374, 24 h: 161.71 ± 2.128; 48 h: 544.69 ± 4.465, 72 h: 230.02 ± 0; *F* (4, 10) = 1177; *P* < 0.0001) of the stressed groups (Figure 9).

## 4. Conclusions

The present study investigates on post stress effects of restraint stress on behavior (anxiety and memory principally) in the context of oxidative stress. The post stress effects have been analyzed previously concerning notably the expression of c-fos [28], BDNF [29], anorexigenic (Pro-opiomelanocortin: POMC and cocaine amphetamine related transcript: CART) and orexigenic (neuropeptide Y:NPY, Agouti related peptide: AgRP) factors in hypothalamus and dorsal vagal complex (DVC) [15]. We also reported on post stress kinetics of orexigenic and anorexigenic neuropeptides expression in two key brain regions hypothalamus and dorsal vagal complex, two key regions involved in food intake regulation using another psychological stress paradigm i.e., immobilization [9],[15]. We evidenced a clear effect on the expression of these neuropeptides up to 72 h post stress. The results obtained in the current study also show noticeable effects due to the application of acute stress (1 hour restraint) using two approaches: behavioral and physiological ones. Thus, it is obvious that stress affects emotional and cognitive functions among stressed mice with long lasting effects up to 72 h after the cessation of the stress.

Generally, 1 h session of stress generates important and significant level of anxiety just after the cessation of the stress. The effect seems to be still more important in the 24 h post stress. This is well evidenced in the case of the OF test, where the number of central squares crossed by stressed animals decreases drastically just after the stress session and is still significantly lower up to 72 h post stress although a tendency in reaching control levels has been observed. A similar scheme is verified concerning time spent in the central squares. Indeed, the fact that when the center of OF is less explored than the periphery of the device indicates that the animal is expressing anxiety. Interestingly, the pattern of the evolution of the scores obtained in OF test coincides with the evolution of oxidative stress markers observed among different brain regions of stressed mice obtained at the four experimental points. In fact, in this study we have analyzed oxidative stress which is defined as an imbalance between the production of radical species (or reactive) oxygen (ROS) and antioxidant cell capacities. The degradation of these radicals is controlled by defense systems, antioxidants, which adapt to the level of the present radicals [30]. ROS have a very short life span, so it is not easy to detect them. Nevertheless, ROS-related tissue destruction could be observed by the final product of lipid peroxidation, such as MDA. Excessive local production and overproduction of ROS lead to increased lipid peroxidation, which is often monitored by measuring the malondialdehyde (MDA) [31],[32]. Modifications in the activity of antioxidative enzymes (catalase, superoxide dismutase, glutathione peroxidase, glutathione reductase), increased malondialdehyde concentration as oxidative stress parameters and the marker of ROS increased level [33]–[35].

In our study, based on the data in the literature concerning the measurement of the quantity of ROS, we measured the quantity of MDA as an indicator of the production rate of these radicals. Our results revealed an increase of MDA which means an overproduction of ROS by the stress as well as disturbances of the antioxidant balance via modifications of the anti-oxidant enzymes (superoxide dismutase, catalase, glutathione peroxidase and glutathione S transferase).

The levels of enzymatic activities of SOD, GPx and GST rise drastically in the hypothalamus compared to other brain regions investigated also in this work. This is highly important as the hypothalamus is the master brain structure involved in the regulation of stress. Indeed, the hypothalamus-pituitary-adrenal glands (HPA) axis is well demonstrated to be the axis of stress [36]. In attempt to alleviate and to attenuate oxidative stress (notably the production of hydrogen peroxide and reactive oxygen) following restraint application, SOD activity rises to counteract the high levels of these products. Of note, SOD and catalase represent the first and important frontline in this operation. As the levels of oxidative stress products rise rapidly, they have in first time down regulated the activities of catalase where SOD takes the relay to counteract them. In a second step, glutathione enzymes, GPX and GST, began to operate. Thus, firstly, the glutathione, which is considered as a strong endogenous antioxidant, is transferred by GST to oxidative stress products to be conjugated to them in order to stop their harmful cellular reactivity. Then, after its conjugation, the glutathione is oxided by GPx. This explains why their activities rise strongly and immediately after stress in hypothalamus.

All these changes are concomitant to the increase of peroxidation level of membranes as indicated by the high levels of lipid peroxidation LPO. Taken together, the results obtained in OF test and those related to oxidative stress markers are in favor of the fact that restraint stress applied acutely results in the long-lasting generation of oxidative stress which leads to homeostasis disturbances, which in turn elicits the occurrence of anxiety state. We also investigated memory skills in the context of acute stress to assess its long lasting effects. In the recognition test, discrimination index reflecting memory and exploratory performance [37] is strongly affected as it decreases strongly up to 72 h. Although a tendency in increase is observed at 72 h post stress, it remains far from the control values. This is of high interest due to the importance of this cognitive faculty. As for the above-mentioned behavioral alteration, the dysfunction of memory must be related to the oxidative stress status. Of note, in the hippocampus, which is one of the important components of memory circuitry, both the enzymatic activities of catalase and SOD i.e. the first frontline of defense (detoxication) are down regulated in parallel to a highly significant increase in lipid peroxidation which in line of the strong down regulation of discrimination index. Also, the activities of GPx and GST are up regulated which indicates their activation by oxidative stress generated by restraint stress.

The high decrease of discrimination index observed in post stress could also be explained by neurochemical dysfunctioning. Acetylcholine is known to be main neurotransmitter involved in memory process [38]–[44]. For this reason, the reduction of AChE may be involved in memory impairment since an excess level of ACh was shown to be neurotoxic. The opposite is also true since low levels of ACh in the synaptic junction may influence memory negatively [45]. According to the literature, acetylcholinesterase inhibitors used in learning are not very powerful, and the level of ACh must not be either deficient or excessive. In our study, AChE was very significantly inhibited in the hippocampus, which can lead to an excess of ACh in the synaptic gaps and have neurotoxic effects on the structures, disrupting their functioning as well as the neurotransmission.

Finally, the case of olfactory bulb (OB) is particularly interesting as this brain structure is related to odor discrimination as well as olfaction memory. Thus, OB presents a similar pattern to that observed in hypothalamus concerning the two antioxidant enzymes of the frontline, but differs in the post stress dynamics of GPx, which are significantly down regulated. This drop is a sign of the fall of the enzyme to counteract the negative effects of restraint stress notably at lasting periods: 48 h and 72h, characterized by an important increase in lipid peroxidation, after early period of resistance. From this, it could be postulated that perturbation of odor discrimination is essential for psychological balance [46]. This could be a part of the genesis of anxiety observed among stressed mice.

In conclusion, the results obtained in this investigation point on the role of oxidative stress in the generation or at least in exacerbating stress effects notably long-lasting effects as the kinetics of oxidative stress biomarkers are very dynamic during a significant post stress period. This corroborates with previous studies among rodents reporting on the post stress deregulation of neurotransmitters/neuromodulators. Thus, one can speculate that the dynamic changes observed in oxidative stress biomarkers could initiate the reported neurochemical changes, which in turn affect behavior.
